# GluN2B protein deficits in the left, but not the right, hippocampus in schizophrenia

**DOI:** 10.1186/s12888-014-0274-z

**Published:** 2014-10-08

**Authors:** Amy E Geddes, Xu-Feng Huang, Kelly A Newell

**Affiliations:** Faculty of Science, Medicine and Health, University of Wollongong, Northfields Ave, Wollongong, NSW 2522 Australia; Illawarra Health and Medical Research Institute, Wollongong, NSW 2522 Australia; Schizophrenia Research Institute, Darlinghurst, NSW 2010 Australia

**Keywords:** GluN2B, NR2B, NMDA receptor, hippocampus, dentate gyrus, human brain

## Abstract

**Background:**

Increasing evidence indicates that alterations to the function and subunit composition of the glutamatergic NMDA receptor are associated with the pathophysiology of schizophrenia. The GluN2B protein is a structural and functional subunit of the NMDA receptor, with a growing body of evidence indicating it plays a critical role in cognitive functions mediated by the NMDA receptor. The hippocampus plays a key role in cognitive function, with studies suggesting lateralised glutamatergic dysfunction in this region may contribute to the cognitive deficits observed in schizophrenia patients. The present study, for the first time, investigated GluN2B protein and binding density in the left and right hippocampus of 20 schizophrenia subjects compared to 20 matched controls.

**Methods:**

The dentate gyrus of 20 schizophrenia and 20 control subjects, matched for age, post-mortem interval, and pH, was obtained from the NSW Tissue Resource Centre, Australia. Each group consisted of dentate gyrus from the left hemisphere (n = 10) and right hemisphere (n = 10). GluN2B protein density was measured via immunoblotting. GluN2B binding density was measured using the GluN2B antagonist, [^3^H] Ifenprodil. Analyses of covariance, covarying for demographic variables that influenced the data, were used to test for statistical significance between schizophrenia and control groups. Pearson’s correlations were used to determine the association of GluN2B protein and binding density with demographic and clinical variables, including lifetime antipsychotic drug exposure.

**Results:**

GluN2B protein levels were decreased by 43% in the left hemisphere of schizophrenia subjects compared to controls (p = 0.012). There was no difference in GluN2B protein levels in the right hemisphere of schizophrenia subjects compared to controls. There were no differences in [^3^H] Ifenprodil binding according to diagnosis or hemisphere. There were no associations between GluN2B measures and lifetime antipsychotic drug exposure.

**Conclusions:**

Our findings provide the first evidence of GluN2 protein abnormalities in the hippocampus in schizophrenia, highlighting the hippocampal lateralisation in this disorder. We suggest this deficit could contribute to the cognitive dysfunctions that arise in patients. These findings provide preliminary support for the development of therapeutics that target the GluN2B subunit, as a novel therapy for schizophrenia, especially the cognitive dysfunctions.

## Background

The hippocampus plays an important role in learning and memory, cognitive functions that are disrupted in schizophrenia [1]. Several lines of research have shown that there are various structural and functional abnormalities in the hippocampus of schizophrenia patients, including a reduction in hippocampal volume, altered synaptic connections (evidenced through reductions in synaptosomal proteins such as synaptophysin, SNAP-25 and synapsin) and changes in neurotransmitter receptors, implicating the hippocampus in the pathophysiology of schizophrenia [2].

Dysregulation of glutamatergic neurotransmission, particularly associated with the N-methyl-D-aspartate glutamate receptor (NMDAR) is also widely believed to play a central role in schizophrenia pathophysiology [3-5]. The NMDAR is assembled in various subunit combinations, with obligatory GluN1 subunits coupled primarily with GluN2A, B, C, or D subunits [6]. Various studies have found alterations in NMDAR binding and GluN1 subunit mRNA and protein in the hippocampus of schizophrenia subjects (For review see [3]). Some replicated findings include a decrease in GluN1 mRNA in the dentate gyrus of schizophrenia patients [7,8], a decrease or no change in NMDAR binding density [7,9,10] and most interestingly a more pronounced decrease in NMDAR binding and GluN1 mRNA and protein in the left hippocampus of schizophrenia subjects compared to the right [8,9,11]. These studies suggest a lateralised glutamatergic dysregulation in the hippocampus of schizophrenia subjects [2]. With regards to the GluN2 subunits, one study has reported an increase in GluN2B mRNA in the hippocampus in schizophrenia, with two further studies reporting no change, and no changes have been reported for GluN2A mRNA, or the less expressed 2C and 2D subunits [7,10,12]. The status of GluN2 proteins in the hippocampus in schizophrenia is currently unknown, however one study did report no change in [^3^H] ifenprodil binding to GluN2B in the hippocampus of schizophrenia subjects [10]. These aforementioned studies, however, did not report hemisphere specific analyses which is an important aspect to consider in studies of the hippocampus [2].

The GluN2 subunit composition defines the functional properties of the NMDAR [3], therefore to understand the NMDAR dysregulation in schizophrenia it is critical to investigate alterations to the GluN2 subunits. GluN2A and GluN2B are the principle subunits in the hippocampus [13]; evidence suggests that the GluN2B subunit in the hippocampus is particularly important for NMDAR channel function [14], long-term potentiation (LTP) and associated cognitive functions such as spatial learning [15]. Accordingly, specifically targeting the GluN2B subunit has been suggested as a novel mechanism to treat cognitive dysfunction in schizophrenia patients and other cognitive disorders [16,17].

The aim of this study was to determine whether there are alterations in GluN2B subunit density in the hippocampus of schizophrenia subjects compared to controls and whether any effects were hemisphere specific. We used immunoblot to detect GluN2B protein density and [^3^H] ifenprodil, which binds to the polyamine site of the GluN2B subunit, to assess GluN2B binding density.

## Methods

### Brain Tissue

Post-mortem human brain tissue was acquired from the NSW Tissue Resource Centre at the University of Sydney. The cohorts consisted of schizophrenia patients, with the diagnosis of schizophrenia confirmed according to DSM-IV and healthy controls, with no known history of psychiatric illness (Table 1). The cohort for the western blotting consisted of 20 schizophrenia and 20 control subjects; the cohort for receptor binding consisted of 20 schizophrenia and 21 control subjects. There was an overlap of 84% (37/44) between subjects included in the western blot and receptor binding studies. There were no significant differences in age, brain pH, post-mortem interval (PMI) or brain weight between the schizophrenia and control subjects included in the protein or binding studies. This study was approved by the University of Wollongong Human Research Ethics Committee (HE99/222).

**Table 1:** **Cohort demographic and clinical characteristics**

**Variable**	**Cohort**	**Control**	**Schizophrenia**	**t**	**p**
n	Western Blot	20	20		
	Receptor binding	21	20		
Age at death	Western Blot	54.5 ± 13.4 (24-73)	54.7 ± 12.8 (27-75)	-0.036	0.971
Mean years ± SD (range)	Receptor binding	55.3 ± 13.1 (24-73)	54.8 ± 12.8 (27-75)	0.132	0.895
Post-mortem interval	Western Blot	28.2 ± 13.3	29.5 ± 12.5	-0.307	0.761
Mean hours ± SD	Receptor binding	30.5 ± 13.6	28.9 ± 12.3	0.388	0.700
pH	Western Blot	6.47 ± 0.35	6.47 ± 0.16	0.069	0.945
Mean ± SD	Receptor binding	6.44 ± 0.35	6.47 ± 0.16	-0.381	0.706
Gender	Western Blot	12 M/8 F	12 M/8 F	N/A	N/A
	Receptor binding	12 M/9 F	12 M/8 F	N/A	N/A
Hemisphere	Western Blot	10 L/10R	11 L/9R	N/A	N/A
	Receptor binding	10 L/11R	10 L/10R	N/A	N/A
Brain weight	Western Blot	1410 ± 128	1358 ± 172	1.085	0.285
Mean grams ± SD	Receptor binding	1396 ± 139	1358 ± 172	0.792	0.433
Estimated daily antipsychotic drug dose	Western Blot	N/A	782 ± 543	N/A	N/A
Mean CPZ equiv ± SD	Receptor binding	N/A	809 ± 529	N/A	N/A
Age of onset	Western Blot	N/A	24.1 ± 8.2 (14-44)	N/A	N/A
Mean years ± SD (range)	Receptor binding	N/A	23.6 ± 8.1 (14-44)	N/A	N/A
Duration of illness	Western Blot	N/A	30.6 ± 11.9 (12-47)	N/A	N/A
Mean years ± SD (range)	Receptor binding	N/A	30.7 ± 11.9 (12-47)	N/A	N/A

There was an overlap of 84% (37/44) between subjects included in the western blot and receptor binding studies. Independent t-tests show no significant differences in age at death, post-mortem interval, pH or brain weight between schizophrenia and control groups; Abbreviations: M, male; F, female; L, left; R, right; PMI, post-mortem interval; CPZ equiv, chlorpromazine equivalents. N/A, not applicable.

### Immunoblot

Immunoblotting was performed as previously described [18]. Tissue from the dentate gyrus of the hippocampus was homogenised in 0.1 M TrisHCl containing 10 μl/ml protease inhibitor cocktail (Sigma-Aldrich), 0.625 μl/ml aprotinin and 0.5 μl/ml glycerol. GluN2B protein density was measured using an anti-GluN2B antibody (#MAB5778, 1/625; Millipore) diluted in PBS-T containing 1% skim milk. A mouse anti-actin antibody was used as an internal standard (#MAB1501, 1/100 000; Millipore). Samples were run in triplicate at a concentration of 10 μg of total protein. GluN2B protein bands were detected on Kodak BioMax MR Film (Sigma-Aldrich) using enhanced chemiliuminescence reagents (GE Healthcare). Films were analysed using Quantity One software (BioRad); the optical density of each band was normalised to the respective β-actin band and averaged over the three runs.

### In situ radioligand binding

[^3^H] Ifenprodil binding was performed based on the method described previously [19]. Three slides per subject, each containing 14 μm hippocampal sections, were incubated for 3 hours at 4°C in 0.05 M TrisHCl (pH 7.4) with 20nM [^3^H] Ifenprodil (GluN2B antagonist: specific activity 40 Ci/mmol; PerkinElmer). Due to previous findings that [^3^H] ifenprodil has some affinity for sigma receptors, piperazine acceptor sites, and non-GluN2B polyamine sites [20-22], the following pharmacological inhibitors were included in the incubation assay: 3 μM R(+)-3-(3-hydroxyphenyl)-N-propylpiperidine hydrochloride (+3PPP; to block non-specific binding to the sigma receptor), 30 μM GBR-12909 (to block piperazine sites) and 1 mM trifluoperazine (to block low affinity polyamine sensitive sites on adrenergic, dopaminergic and cholinergic receptors). Adjacent sections were incubated in the same solution, with the addition of 20 μM Ifenprodil (Sigma-Aldrich) to determine any non-specific binding. All sections were washed in 0.05 M TrisHCl (pH 7.4) for 3x5 minutes at 4°C, rinsed in distilled water and air-dried. All slides were exposed to Kodak BioMax MR Film (Sigma-Aldrich) for 16 weeks. Films were analysed using Quantity One software; quantification was performed by taking the average optical density from the dentate gyrus in three slides. Specific binding was determined by subtracting non-specific binding density from total binding density.

### Statistical Analyses

The data were checked for triplicate (standard deviation > 30% mean) and population outliers (using box-plots) and Kolmogorov-Smirnov tests were used to confirm data normality. T-tests, ANOVAs and ANCOVAs with LSD post-hoc were used to determine the effects of diagnosis overall and within the left and right hemispheres, co-varying for variables that were shown to correlate with the data. Pearson’s correlations were used to determine relationships to continuous variables including age at death, PMI, pH, brain weight, antipsychotic medication, age of illness onset, and duration of illness.

## Results

### Left hippocampal deficits in GluN2B subunit protein in schizophrenia subjects

**Figure 1:**
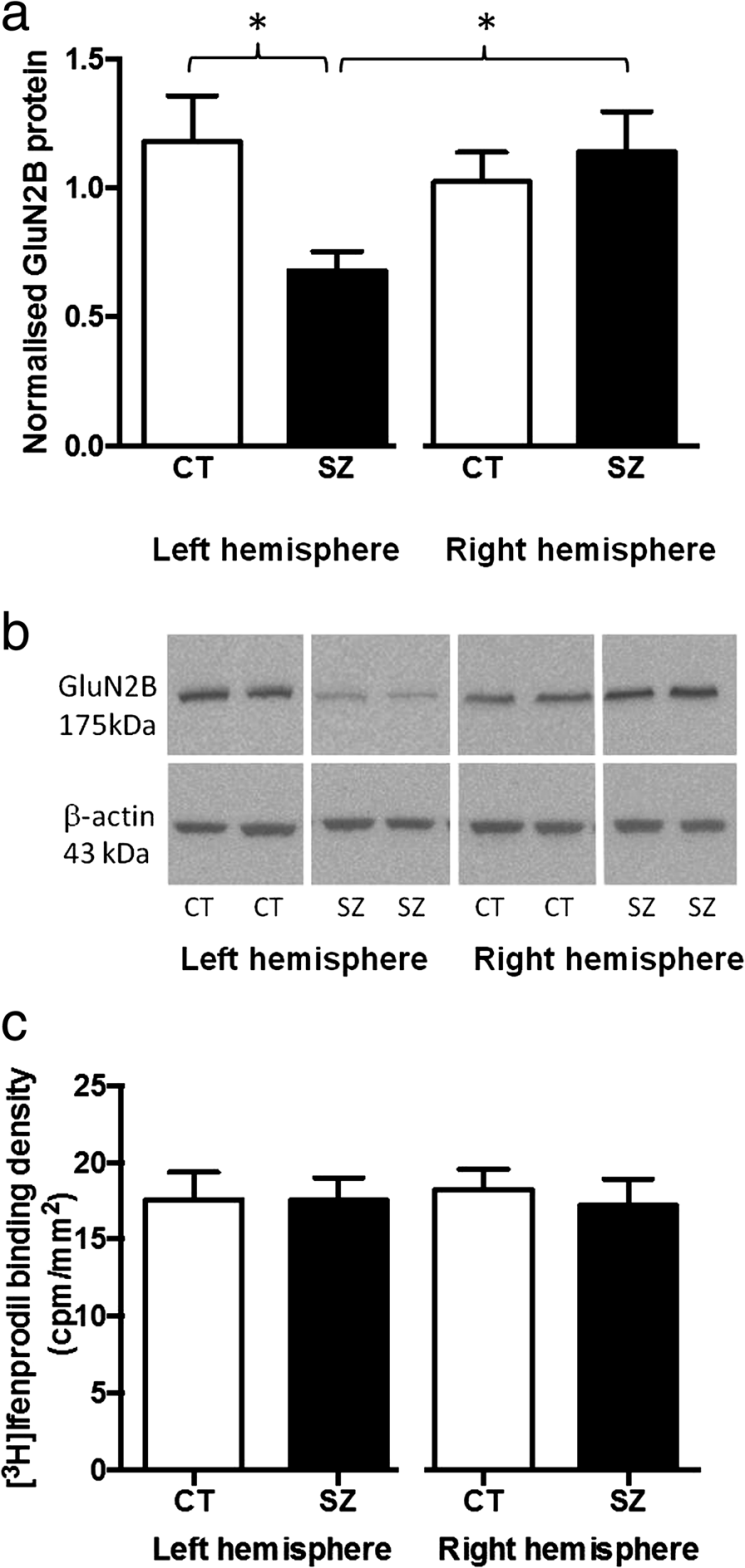
**GluN2B protein and binding density in the hippocampus (dentate gyrus) of schizophrenia and control subjects. a**. GluN2B protein density, normalised to β-actin, in the left and right hemisphere of schizophrenia (SZ) and control (CT) subjects. GluN2B was reduced in the left hemisphere of subjects with schizophrenia, compared to the left hemisphere of controls (−43%, p = 0.012) and compared to the right hemisphere of schizophrenia subjects (−40%, p = 0.023 ). *:p < 0.05. **b**. Representative blot showing GluN2B and β-actin protein expression in control (CT) and schizophrenia subjects (SZ) from the left and right hemisphere. GluN2B was identified as a single band at the expected molecular weight (175 kDa). **c**. [^3^H] Ifenprodil binding to GluN2B revealed no significant differences between schizophrenia (SZ) and control (CT) subjects, in the left or right hemisphere. Bars represent mean + SEM.

While we observed no overall difference in GluN2B protein density in the dentate gyrus of schizophrenia subjects compared to controls (t_36_ = 1.452, p = 0.155), we found a significant difference between hemisphere-specific sub-groups (F_3,34_ = 2.882, p = 0.050), with a 43% reduction in GluN2B protein in the left hippocampus of schizophrenia subjects compared to the left hippocampus of controls (p = 0.012) and a 40% reduction in GluN2B protein in the left hippocampus of schizophrenia subjects compared to the right hippocampus from schizophrenia subjects (p = 0.023) (Figure 1a and 1b). GluN2B protein density correlated significantly with brain pH (r = −0.386, p = 0.017) and there was a trend for a correlation with PMI (r = −0.318, p = 0.051) in the whole cohort (Table 2). Similar correlations were observed in the control subjects (pH: r = −0.662, p = 0.002; PMI: r = −0.483, p = 0.036) but not the schizophrenia subjects (pH: r = 0.162, p = 0.506; PMI: r = −0.116, p = 0.636). ANCOVAs correcting for brain pH and PMI confirmed that there was no overall difference in GluN2B protein density between schizophrenia and control subjects (F_1,34_ = 2.004, p = 0.166) and that there was a significant effect between the 4 hemisphere-specific subgroups (F_3,32_ = 3.824, p = 0.019). Importantly, there was no correlation between GluN2B protein density in the schizophrenia subjects and length of illness, age of disease onset or estimated daily antipsychotic medication dose (Table 2).

**Table 2:** **Pearsons correlations for continuous variables influencing GluN2B binding and protein expression in the hippocampus of all subjects, control subjects only and schizophrenia subjects only**

**Variable**		**All subjects**		**Control**		**Schizophrenia**	
		**GluN2B**	**GluN2B**	**GluN2B**	**GluN2B**	**GluN2B**	**GluN2B**
		**Protein**	**Binding**	**Protein**	**Binding**	**Protein**	**Binding**
n		38	41	19	21	19	20
Age at death	r	0.16	**‐0.309**	0.207	-0.408	0.152	-0.212
	p	0.338	**0.049**	0.395	0.067	0.534	0.369
Post-mortem interval	r	**‐0.318**	0.054	**‐0.483**	0.061	-0.116	0.030
	p	**0.051**	0.738	**0.036**	0.794	0.636	0.901
pH	r	**‐0.386**	0.068	**‐0.662**	0.226	0.162	-0.255
	p	**0.017**	0.671	**0.002**	0.324	0.506	0.278
Brain weight	r	0.067	-0.029	0.028	0.152	0.023	-0.218
	p	0.688	0.855	0.908	0.512	0.926	0.356
Duration of illness	r	N/A	N/A	N/A	N/A	-0.056	-0.192
	p	N/A	N/A	N/A	N/A	0.819	0.430
Age of onset	r	N/A	N/A	N/A	N/A	0.313	0.007
	p	N/A	N/A	N/A	N/A	0.193	0.977
Estimated daily antipsychotic drug dose	r	N/A	N/A	N/A	N/A	-0.156	-0.368
	p	N/A	N/A	N/A	N/A	0.525	0.111

Significant and borderline significant correlations are highlighted in bold. N/A, not applicable.

### No change in GluN2B binding density in schizophrenia subjects compared to controls

There was no overall difference in [^3^H] Ifenprodil binding in the dentate gyrus between schizophrenia and control subjects (t_39_ = 0.808, p = 0.424) and no hemisphere dependent effects (F_3,37_ = 0.394, p = 0.758) (Figure 1c). [^3^H] Ifenprodil binding density correlated with age at death in the whole cohort (r = −0.309, p = 0.049), showed a trend towards a correlation in control subjects (r = −0.408, p = 0.067), but did not correlate in schizophrenia subjects (r = −0.212, p = 0.369) (Table 2). ANCOVAs to correct for age at death confirmed no difference in [^3^H] Ifenprodil binding density between schizophrenia and control subjects (F_1,38_ = 0.780, p = 0.383) or hemisphere-specific subgroups (F_3,36_ = 0.472, p = 0.704). There was no correlation between GluN2B binding density in the schizophrenia subjects and length of illness, age of disease onset or estimated daily antipsychotic medication dose (Table 2).

## Discussion

In the present study we have identified a large (43%) reduction in GluN2B protein levels in the hippocampal dentate gyrus in schizophrenia. This reduction was specific to the left hemisphere, highlighting the hippocampal lateralisation in the pathophysiology of schizophrenia. In addition, we have confirmed previous results, this time in a larger cohort (n = 15 cf. n = 20), indicating no change in [^3^H] ifenprodil binding to GluN2B in the hippocampus of schizophrenia subjects in comparison to controls [10] and extended these findings to report no hemisphere specific alteration in GluN2B binding density.

It is well documented that the hippocampus is altered in schizophrenia and there is evidence to suggest that the left hemisphere shows more pronounced deficits in NMDAR expression than the right hemisphere. Previous studies have reported a more pronounced decrease in GluN1 mRNA and protein in the left hemisphere of schizophrenia subjects [8,11] as well as a reduction in [^123^I] CNS-1261 binding to the NMDAR channel in the left hippocampus of medication free schizophrenia subjects [9]. Ours is the first study in the hippocampus to report such a lateralisation effect with any of the GluN2 subunits, however, to our knowledge, the protein density of the other GluN2 subunits has not yet been investigated in this region. It has previously been reported that deletion of the GluN1 subunit in the CA1 region of the hippocampus results in reduced expression of the GluN2B subunit in the dendrites of pyramidal neurons in mice [23], suggesting our finding of reduced GluN2B in the hippocampus could be associated with the reductions in GluN1 previously reported in the left hippocampus in schizophrenia.

While we found a reduction in GluN2B protein levels in the present study, this was accompanied by no change in GluN2B binding density. [^3^H] Ifenprodil binds to the polyamine site on GluN2B subunits. While ifenprodil does also have some affinity for sigma, piperazine, adrenergic receptors and other polyamine sites [20-22], pharmacological inhibitors were used to block these sites in the binding assay. Therefore, it is unlikely that the discrepancy between binding and protein is a result of [^3^H] Ifenprodil binding to non-GluN2B sites. It is possible however that that these two techniques label different populations of GluN2B subunits. Under the conditions used in the present study (i.e. the use of tissue sections rather than cell lysed homogenates), [^3^H] ifenprodil would bind primarily to cell surface receptors, while the immunoblot would detect GluN2B protein in all cellular compartments. This suggests that the reduction observed in GluN2B protein in the present study might represent a reduction in intracellular protein levels. The implications of such localised reductions remain unclear [24]. Similar to our study, in cortical brain areas it has been shown that despite no change in [^3^H] ifenprodil binding [25], there was a reduction in GluN2B protein in schizophrenia compared to control subjects, which was specific to the endoplasmic reticulum [26], suggesting there may be altered production, degradation or trafficking of GluN2B protein in schizophrenia. While we did not examine GluN2B mRNA in the present study, two previous studies reported no change in GluN2B mRNA levels in the dentate gyrus in schizophrenia [10,12], therefore it is unlikely the findings in the present study represent changes in GluN2B production. It is possible however that our findings reflect disturbances to GluN2B trafficking, degradation, or other aspects of GluN2B regulation. Further studies, specifically examining isolated cellular compartments and proteins that regulate trafficking/degradation of GluN2B, as well as assessments of GluN2B phosphorylation and activation would be important to further understand the GluN2B alterations in the hippocampus in schizophrenia.

GluN2B containing NMDARs play a critical role in cognitive functions, especially in the hippocampus (For review see [27]). Selective reduction of GluN2B-containing NMDARs in the hippocampus has been reported to produce cognitive deficits in rodents [15,28] as well as disrupting underlying molecular processes such as LTP [15]. Specifically, the left hippocampus has previously been associated with verbal and visual memory performance in schizophrenia subjects [29]. Therefore, a reduction in NMDARs or altered composition of the NMDAR subunits in hippocampal subregions, such as that found in the present study, may be a contributing factor to the cognitive deficits observed in these patients. Therapies aiming to enhance GluN2B function or activity have been shown to improve cognitive function in animal models of ageing-induced cognitive impairment [30]. Similarly, over-expression of GluN2B in transgenic mice results in increased cognitive ability [31]. Along these lines, positive allosteric modulation, selective for GluN2B-containing NMDARs has been proposed as a novel therapeutic strategy for the treatment of schizophrenia and cognitive dysfunction more generally [16,17]. Our finding therefore of reduced GluN2B protein in the hippocampus, is likely to contribute to the cognitive symptoms in schizophrenia patients, and supports the idea that GluN2B positive allosteric modulators could potentially be therapeutic for cognitive dysfunctions in schizophrenia.

As with all post-mortem human brain studies of this nature, we cannot rule out an influence of ante-mortem medication on our data. However, we observed no correlation between antipsychotic dose (measured as chlorpromazine equivalents) and GluN2B binding or protein levels. Furthermore, there is evidence from animal studies that antipsychotic drug treatment does not alter GluN2B protein levels in the brain [32-34], suggesting that our findings are unlikely to be due to the effects of pre-mortem antipsychotic drug treatment.

## Conclusion

In conclusion, the present study for the first time revealed that GluN2B protein levels are significantly decreased specifically in the left hippocampus (dentate gyrus) of schizophrenia subjects compared to controls. We suggest this reduction may be associated with cognitive symptoms arising in schizophrenia subjects. While further studies are warranted to determine the functional implications of this reduction and if this reduction is common to other GluN2 subunits or hippocampal subregions, our findings provide preliminary support for the development of GluN2B selective positive modulators as a novel therapy for schizophrenia, especially the cognitive dysfunctions.

## Abbreviations

LTP: long-term potentiation
NMDAR: N-methyl-D-aspartate receptor
PMI: post-mortem interval
